# Low-level alcohol consumption and cancer mortality

**DOI:** 10.1038/s41598-021-84181-1

**Published:** 2021-02-25

**Authors:** Hyeonyoung Ko, Yoosoo Chang, Han-Na Kim, Jae-Heon Kang, Hocheol Shin, Eunju Sung, Seungho Ryu

**Affiliations:** 1 Department of Family Medicine, Kangbuk Samsung Hospital, Sungkyunkwan University School of Medicine, 29 Saemunan-ro, Jongno-gu, Seoul, 03181 Republic of Korea; 2 Samsung Seocho Medical Clinic, Kangbuk Samsung Hospital, Sungkyunkwan University School of Medicine, Seoul, Republic of Korea; 3 Center for Cohort Studies, Total Healthcare Center, Kangbuk Samsung Hospital, Sungkyunkwan University School of Medicine, Seoul, Republic of Korea; 4 Department of Occupational and Environmental Medicine, Kangbuk Samsung Hospital, Sungkyunkwan University School of Medicine, Samsung Main Building B2, 250 Taepyung-ro 2ga, Jung-gu, Seoul, 04514 Republic of Korea; 5 Department of Clinical Research Design & Evaluation, SAIHST, Sungkyunkwan University, Seoul, Republic of Korea; 6 Medical Research Institute, Kangbuk Samsung Hospital, Sungkyunkwan University School of Medicine, Seoul, Republic of Korea

**Keywords:** Cancer, Risk factors

## Abstract

The effect of light-to-moderate alcohol consumption on cancer risk remains controversial. We examined the association between low-level alcohol consumption and cancer mortality. A cohort study included 331,984 Korean adults free of cancer at baseline who underwent a comprehensive health checkup examination. Participants were categorized into never drinkers, former drinkers, and current drinkers who were further divided into light, moderate, heavy, and very heavy drinkers. Vital status and cancer-related deaths were ascertained through links to national death records. During 1,633,906 person-years of follow-up (median 5.3 years interquartile range 3.8–6.2), 374 cancer-related deaths were identified (cancer-cause mortality rate of 23 per 10^5^ person-years). When former and never drinkers were classified as non-drinkers, the light drinkers had a lowest risk of cancer mortality compared with non-drinkers and other current drinkers (J-shaped); however, with consideration of lifetime abstinence history, current drinking was positively associated with cancer mortality in a dose-dependent manner. When changes in alcohol drinking status and confounders during follow-up were updated as time-varying covariates and never drinkers were used as the reference, the multivariable-adjusted hazard ratios (HRs) (95% confidence intervals, CIs) for cancer mortality among current light, moderate, heavy, and very heavy drinkers were 1.58 (1.03–2.43), 2.28 (1.41–3.70), 2.34 (1.42–3.85), and 2.97 (1.80–4.90), respectively, and the highest risk of cancer mortality was observed in former drinkers, who had an HR (95% CI) of 3.86 (2.38–6.28). Alcohol consumption was significantly and positively associated with an increased risk of cancer mortality in a dose-dependent manner, beginning with light drinkers.

## Introduction

Alcohol consumption is one of the most important known and modifiable risk factors for cancers^1^. In 2012, 5.5% of all cancer cases and 5.8% of all cancer deaths worldwide were attributable to alcohol consumption^2^. A body of evidence suggests that alcohol consumption increases the risk of cancers in the oral cavity, pharynx, larynx, esophagus, liver, breast, and colorectum in a dose-dependent manner beginning with low and moderate drinking^1,3–5^.

However, the effect of light-to-moderate drinking on cancer risk and mortality remains controversial. Multiple cohort studies and meta-analyses have reported a J-shaped relationship between alcohol drinking and cancer mortality, with the lowest risk seen in light-to-moderate drinkers^6–9^. However, the studies that show beneficial health effects from low-volume drinking have typically misclassified former drinkers as abstainers, lacked lifetime drinking history, made insufficient adjustment for potential confounders, or studied only middle-aged or elderly adults^10–12^. Former drinkers might currently abstain for health reasons (sick quitter hypothesis) and thus be at high risk for short-term mortality^10,13^; however, most studies have included former drinkers in the reference group and thus did not provide a valid comparison between people who have consumed alcohol and those who have never drunk it. Furthermore, most studies have used a single measure of alcohol consumption at one time point although alcohol intake can change over time^14^.

Therefore, we examined whether alcohol consumption was associated with cancer-related mortality in a cohort of 331,984 young and middle-aged Korean men and women, free of cancer at baseline, who participated in a health screening examination program. We accounted for lifetime abstinence history and used time-dependent measures of change in alcohol drinking status and other covariates during follow-up.

## Methods

### Study population

The Kangbuk Samsung Health Study is an ongoing cohort study of Korean men and women who regularly participated in comprehensive health examinations at one of the Kangbuk Samsung Hospital Total Healthcare Centers in Seoul and Suwon, South Korea as described elsewhere^15^ (See the further detail in the supplementary file and Supplementary Table 1). Over 80% of participants were employees of various companies and local governmental organizations and their spouses and are relatively highly educated, young to middle-age Korean adults with high accessibility to health care resources. In South Korea, the Industrial Safety and Health Law requires annual or biennial health screening exams of all employees, offered free of charge. The present study population was restricted to Kangbuk Samsung Health Study participants who underwent a comprehensive health screening exam between 2011 (when lifetime abstinence history was added as part of the health questionnaire) and 2015 (*N* = 371,550) and were followed through the end of 2017.

Out of 371,550 participants, we excluded 39,566 subjects (10.6%) who met the following exclusion criteria at baseline: unknown vital status due to invalid identifier (*n* = 1), missing data on alcohol drinking history (*n* = 31,780), missing data on BMI (*n* = 489) and having a history of malignancy (*n* = 9,150). Because some participants met more than one exclusion criterion, 331,984 participants were eligible for this study.

### Data collection

Data regarding demographic characteristics, behavioral factors, dietary intake, and medical history were collected using a standardized, self-administered questionnaire. Participants were categorized by smoking status as never, former, or current smokers. Physical activity was assessed using the validated Korean version of the International Physical Activity Questionnaire short form^16^, and participants were classified into one of three categories: inactive, minimally active, or health-enhancing physical activity (HEPA)^16^. HEPA was defined as physical activity that meets either of two criteria: (i) vigorous intensity activity on three or more days per week accumulating ≥ 1500 MET min/week, or (ii) seven days with any combination of walking, moderate intensity, or vigorous intensity activities achieving at least 3000 MET min/week^16^.

Sitting blood pressure (BP), height, and weight were measured by trained nurses. Obesity was defined as a body mass index (BMI) ≥ 25 kg/m^2^, the proposed cutoff for a diagnosis of obesity in Asians^17^. Hypertension was defined as systolic blood pressure ≥ 140 mmHg, diastolic blood pressure ≥ 90 mmHg, or the use of antihypertensive medication. Fasting blood tests included total cholesterol, low-density lipoprotein cholesterol (LDL-C), high-density lipoprotein cholesterol (HDL-C), triglycerides, glucose, insulin, high sensitivity-C reactive protein (hsCRP), and liver enzymes^18^. Insulin resistance was assessed using the homeostatic model assessment of insulin resistance (HOMA-IR) equation as follows: fasting blood insulin (uU/mL) × fasting blood glucose (mmol/L)/22.5. Diabetes mellitus was defined as a fasting serum glucose level ≥ 126 mg/dL, hemoglobin A1c ≥ 6.5%, or the current use of anti-diabetic medication. The Laboratory Medicine Department at the Kangbuk Samsung Hospital in Seoul, Korea has been accredited by the Korean Society of Laboratory Medicine (KSLM), the Korean Association of Quality Assurance for Clinical Laboratories (KAQACL) and the CAP (Collage of American Pathologists) Survey Proficiency Testing^18,19^.

### Definition of alcohol consumption

Never drinkers were defined as those who never drank in their lives besides drinking a ritual sip during certain ceremonies. Former drinkers were defined as those with a previous history of drinking alcohol who were non-drinkers at the time of examination. Current alcohol use was assessed as the frequency of alcohol consumption and the amount of alcohol consumed per drinking day using the following questions^20,21^: “How often do you drink alcohol in a week on average (please fill in the details that apply to your current situation)?” and “How much alcohol do you usually drink per drinking day?”. Amount of alcohol consumed per drinking day was recorded in units of ‘soju,’ which is the most popular alcoholic beverage in Korea. The alcoholic content in *soju* was estimated to be 20% at the time of enrollment and the traditional unit of *jan* of soju (1 *jan* of *soju* = 50 mL) contained 8 g of ethanol^21^. The questionnaire listed the volume of alcoholic beverages that contained the equivalent quantity of ethanol to one unit (*jan)* of soju, and the subjects referred to this chart when recording the alcohol amount consumed per drinking day^21^.

Average alcohol consumption per day was calculated using the frequency and amount of alcohol consumed per drinking day. First, alcohol drinking status based on current alcohol consumption was classified as light drinking, moderate drinking, heavy drinking, and very heavy drinking (0.1–< 10, 10–< 20, 20–< 40, ≥ 40 g/day for women, respectively, and 0.1–< 10, 10–< 30, 30–< 60, ≥ 60 g/day for men)^22,23^. Then, with consideration of lifetime abstinence history, participants were classified as never drinkers, former drinkers, light drinkers, moderate drinkers, heavy drinkers and very heavy drinkers. Information on alcohol consumption was annually or biannually collected as part of the basic health check-up program at baseline and follow-up visits.

### Ascertainment of cancer mortality

Mortality follow-up until the end of 2017 was based on nationwide death certificate data from the Korea National Statistical Office. The cause of death was determined based on the underlying cause listed on each death certificate, which was classified according to the International Classification of Diseases and Related Health Problems 10th Revision (ICD-10). Concordance between the cause of death on the death certificate and patient diagnosis in the medical utilization data was 94.9% for cancer deaths^24^. Cancer related mortality was defined as ICD-10 codes C00 to C97. Cancer-related deaths were further divided into alcohol-related cancer deaths and other cancer deaths. Alcohol-related cancers were defined as cancer of the colorectum, female breast, oral cavity (cancers of the salivary gland, codes C07–C08, were excluded from alcohol-related cancers because of their different etiologic patterns compared with other oral cavity cancers)^25^, pharynx, larynx, liver, and esophagus, following the International Agency for Cancer Research^5^.

### Statistical analysis

The characteristics of study participants at baseline are presented by alcohol consumption category. All data are presented as the mean (standard deviation, SD), median (interquartile range), or number (percentage), as appropriate. Baseline characteristics of study participants was compared according to the availability of alcohol information. For a clear comparison between people who have consumed alcohol and those who have never drunk it^10,13^, we differentiated never drinkers from former drinkers and used never drinkers as a reference group.

Each participant was followed from the baseline exam until either the development of cancer-related mortality or December 31, 2017, whichever came first. Participants who died of causes other than cancer before end of 2017 were censored at the date of death. Hazard ratios (HRs) and 95% confidence intervals (CIs) for cancer mortality were estimated using Cox-proportional hazard regression analyses with age as the timescale. The proportional hazards assumption was assessed by examining graphs of the estimated log [− log (*SURVIVAL*)]. In addition to categorical analysis, we modeled alcohol consumption as restricted cubic splines with knots at the at the 5th, 27.5th, 50th, 72.5th, and 95th percentiles of alcohol consumption distribution to provide a flexible estimate of the concentration–response relationship between alcohol consumption and risk of cancer mortality.

Analyses were performed separately for men and women because the association between alcohol consumption and cancer mortality differed marginally by sex. Models were initially adjusted for age (timescale) and sex (for total subjects) and then further adjusted for study center, year of screening exam, smoking, physical activity level, BMI, education level, total energy intake (quintile or unknown), history of hypertension, history of diabetes, history of cardiovascular disease (CVD), and family history of cancer. We conducted time-dependent analyses in which changes in alcohol drinking status and covariates during follow-up were updated as time-varying covariates in the models.

The effect of alcohol consumption on the various health outcomes can depend on the drinking pattern, frequency and intensity of alcohol consumption and a binge drinking pattern also may increase cancer risk, independent of the overall amount of alcohol consumed^26,27^. Thus, we performed additional analyses to investigate the associations between drinking frequency and also alcohol quantity consumed per drinking day with the risk of cancer mortality.

Additionally, we conducted a competing risk analysis using cancer mortality and other cause mortality as competing events according to the method of Fine and Gray to account for the competing risk of death^5,25^. We performed 3-year lag time analyses by excluding cancer mortality cases that occurred within the first 3 years to deal with possibility in which cancer was present but undiagnosed. Stratified analyses were performed by age (< 40 years old vs ≥ 40 years old). Because cancer mortality is associated with smoking, which correlated with alcohol intake, we performed the sensitivity analyses among never smokers to address the residual confounding by smoking.

Statistical analyses were performed using STATA version 15.0 (StataCorp LP, College Station, TX, USA). All reported *P* values are two-tailed. Differences with a *P* value < 0.05 were considered statistically significant.

### Ethical approval

The present study does not include any animal studies. All procedures involved in this study of human participants were in accordance with the ethical standards of the institutional research committee and with the 1964 Helsinki declaration and its later amendments or comparable ethical standards. The general protocol used for a retrospective cohort study (KSHS) was approved by the Institutional Review Board of Kangbuk Samsung Hospital and has been updated on a yearly basis since 2011 (IRB No. KBSMC 2011-01-030-005). Specifically the present study was approved by the Institutional Review Board of Kangbuk Samsung Hospital (KBSMC 2019-05-019) witch waived the need for informed consent due to the use of preexisting, anonymized, retrospective data that were routinely collected during the health-screening process and linked to mortality data from the Korea National Statistical Office^28–30^. Human participants' names and other HIPAA identifiers were not used during the study process and were not included in all sections of the manuscript, including Supplementary Information.

## Results

At baseline, the mean (SD) age was 38.9 (9.4) years and 56.2 percent of the participants were male (Table 1). The alcohol consumption category was positively associated with being male, current smoking status, physical activity, obesity, BMI, BP, total cholesterol LDL-C, glucose, triglycerides, liver enzymes, HOMA-IR, and hsCRP (Table 1, Supplementary Tables 2 and 3). Never drinkers were more likely to be older and women and to have hypertension, diabetes, and CVD and less likely to be highly educated than former drinkers.

**Table 1:** Baseline characteristics according to drinking status (n = 331,984).

Characteristics	Overall	Drinking status					
		Never drinkers	Former drinkers	0.1 to < 10 g/day	10 to < 20 g/day	20 to < 40 g/day	≥ 40 g/day
Number	331,984	18,455	27,115	148,310	58,270	43,608	36,226
Age (years)^a^	38.9 (9.4)	47.0 (12.4)	37.6 (8.8)	37.8 (8.8)	38.3 (8.8)	39.6 (9.2)	40.3 (9.2)
Male (%)	56.2	16.4	29.9	42.0	74.6	84.7	90.7
Current smoker (%)	23.8	4.7	7.7	14.1	30.5	42.5	49.7
High education level (%)^c^	80.0	62.5	78.9	82.6	82.5	80.0	75.5
HEPA (%)^d^	16.6	16.5	14.2	15.2	16.8	18.9	20.7
Hypertension (%)	11.8	16.6	6.9	7.8	12.5	17.3	21.7
Diabetes (%)	3.8	5.7	2.5	2.5	3.7	5.4	7.2
History of CVD (%)	13.2	13.5	10.6	12.2	13.5	15.0	16.3
Family history of cancer (%)	27.4	31.3	22.8	26.9	27.5	28.1	29.9
Obesity (%)^e^	28.2	22.0	20.0	21.4	33.0	39.1	44.7
Body mass index (kg/m^2^)^a^	23.3 (3.3)	22.7 (3.3)	22.4 (3.4)	22.6 (3.3)	23.8 (3.2)	24.3 (3.1)	24.8 (3.1)
Systolic BP (mmHg)^a^	109.6 (13.3)	108.4 (14.0)	104.2 (12.7)	106.8 (12.6)	111.8 (12.6)	114.4 (12.6)	116.4 (12.5)
Diastolic BP (mmHg)^a^	70.2 (10.0)	68.3 (9.4)	66.8 (9.3)	68.1 (9.4)	71.7 (9.7)	73.9 (10.0)	75.5 (10.0)
Glucose (mg/dL)^a^	95.4 (15.1)	95.5 (16.4)	92.3 (13.4)	93.5 (13.0)	96.1 (15.0)	98.5 (17.2)	100.7 (18.9)
Total cholesterol (mg/dL)^a^	193.9 (34.4)	195.6 (35.7)	189.6 (33.6)	190.5 (33.5)	195.8 (34.1)	198.8 (34.5)	201.1 (35.6)
LDL-C (mg/dL)^a^	119.7 (32.1)	121.6 (33.1)	114.0 (30.9)	116.9 (31.4)	122.6 (32.1)	124.2 (32.4)	124.5 (33.0)
HDL-C (mg/dL)^a^	58.5 (15.2)	60.0 (15.0)	60.4 (15.0)	59.9 (15.0)	56.6 (15.0)	56.2 (14.9)	56.5 (15.0)
Triglycerides (mg/dL)^b^	91 (64–136)	84 (61–119)	77 (57–111)	81 (59–117)	99 (69–146)	112 (77–165)	123 (85–182)
AST (U/L)^b^	20 (16–25)	19 (16–24)	18 (16–22)	19 (16–23)	20 (17–25)	22 (18–27)	23 (19–29)
ALT (U/L)^b^	18 (13–27)	16 (12–22)	15 (11–22)	16 (12–24)	20 (14–30)	22 (16–33)	24 (17–35)
GGT (U/L)^b^	21 (13–36)	15 (11–22)	15 (11–23)	16 (12–26)	25 (16–40)	32 (21–54)	43 (26–74)
hsCRP (mg/L)^b^	0.4 (0.2–0.9)	0.4 (0.2–0.9)	0.4 (0.2–0.9)	0.4 (0.2–0.8)	0.5 (0.2–1.0)	0.5 (0.3–1.0)	0.5 (0.3–1.1)
HOMA-IR^b^	1.18 (0.78–1.77)	1.15 (0.75–1.73)	1.13 (0.75–1.67)	1.13 (0.75–1.68)	1.21 (0.8–1.82)	1.26 (0.83–1.89)	1.31 (0.85–1.99)
Total calorie intake (kcal/day)^b,f^	1553.7 (1197.9–1949.4)	1468.8 (1118.3–1843.4)	1458.6 (1086.5–1861.2)	1521.4 (1169.6–1903.9)	1581.2 (1242.3–1972.4)	1619.3 (1269.3–2026.5)	1682.5 (1317.6–2119.6)

Data are expressed as ^a^mean (standard deviation), ^b^median (interquartile range), or percentage. ^c^ ≥ College graduate, ^d^Defined as physical activity that meets either of two criteria: (i) vigorous-intensity activity on three or more days per week accumulating ≥ 1500 metabolic equivalent (MET) min/week; or (ii) seven days of any combination of walking, moderate-intensity, or vigorous-intensity activities achieving at least 3000 MET-min/week; ^e^body mass index ≥ 25 kg/m^2^. ^f^Among 227,464 participants with plausible estimated energy intake levels (within three standard deviations of the log-transformed mean energy intake).

*ALT* alanine aminotransferase, *AST* aspartate aminotransferase, *BP* blood pressure, *CVD* cardiovascular disease, *GGT* gamma-glutamyl transpeptidase, *HDL-C* high-density lipoprotein-cholesterol, *HEPA* health-enhancing physically active, *hsCRP* high sensitivity C-reactive protein, *HOMA-IR* homeostasis model assessment of insulin resistance, *LDL-C* low-density lipoprotein cholesterol.

During 1,633,906.4 person-years of follow-up, 889 all cause deaths (all cause mortality rate of 54.4 per 10^5^ person-years) and 374 cancer-related deaths were identified (42.1% of all deaths; and cancer-cause mortality rate of 23 per 10^5^ person-years). The median duration of follow-up was 5.3 years (interquartile range, 3. 8–6.2 years). The median number of follow-up visits per participant was 2 (interquartile range, 1–3). The top 3 causes of cancer deaths were lung, hepatobiliary and pancreatic cancer in that order for men, and pancreatic, stomach and lung cancer in that order for women, respectively (Supplementary Table 4). When consideration of lifetime abstinence history, drinking categories were positively associated with cancer mortality in a dose-dependent manner, beginning with light drinking (Table 2). Compared with the never drinkers, the age- and sex-adjusted HRs (95% CIs) for cancer mortality among light, moderate, heavy, and very heavy drinkers were 1.67 (1.09–2.54), 2.41 (1.49–3.87), 2.66 (1.64–4.31) and 2.88 (1.76–4.72), respectively. After further adjustment for center, year of screening exam, smoking status, total energy intake, physical activity, BMI, education level, history of diabetes, history of hypertension, history of cardiovascular disease, and family history of cancer, the association between alcohol consumption and cancer mortality remained significant. The association did not differ by sex (p for interaction by sex = 0.257), but light drinking is not associated with cancer mortality in men. In the time-dependent model using never drinkers as the reference, the multivariable-adjusted HRs (95% CIs) for cancer mortality among light, moderate, heavy, and very heavy drinkers were 1.58 (1.03–2.43), 2.28 (1.41–3.70), 2.34 (1.42–3.85), and 2.97 (1.80–4.90), respectively, and the highest risk of cancer mortality was observed in former drinkers, who had an HR (95% CI) of 3.86 (2.38–6.28). In spline models, the risk of cancer mortality started to increase at the light drinking level (Supplementary Fig. 1). When mortality from alcohol-related cancers vs. other cancers was analyzed separately (Supplementary Table 5), the association was stronger for alcohol-related cancer mortality.

**Table 2:** Hazard ratios (95% CI) for cancer mortality by alcohol consumption category based on both lifetime abstinence history and current drinking status (*n* = 331,984).

Alcohol consumption category	Median (IQR) alcohol consumption (g/day)	Person-years	Number of events	Mortality rate (100,000 person-years)	Age-adjusted HRs (95% CI)	Multivariable-adjusted HR (95% CI)^a^	HR (95% CI)^b^ in model using time-dependent variables
**Total (n = 331,984)**							
Never drinker	0 (0–0)	87,360.7	30	34.3	1.00 (reference)	1.00 (reference)	1.00 (reference)
Former drinker	0 (0–0)	134,645.6	33	24.5	2.75 (1.65–4.57)	2.89 (1.73–4.81)	3.86 (2.38–6.28)
0.1 to < 10 g/day	4 (2–6)	722,653.1	114	15.8	1.67 (1.09–2.54)	1.70 (1.11–2.59)	1.58 (1.03–2.43)
10 to < 20 g/day	14 (10–14)	291,163.3	68	23.4	2.41 (1.49–3.87)	2.39 (1.48–3.85)	2.28 (1.41–3.70)
20 to < 40 g/day	27 (23–32)	218,459.4	67	30.7	2.66 (1.64–4.31)	2.57 (1.57–4.19)	2.34 (1.42–3.85)
≥ 40 g/day	55 (46–71)	179,624.4	62	34.5	2.88 (1.76–4.72)	2.74 (1.67–4.52)	2.97 (1.80–4.90)
**Men (n = 186,651)**							
Never drinker	0 (0–0)	14,397.5	10	69.5	1.00 (reference)	1.00 (reference)	1.00 (reference)
Former drinker	0 (0–0)	42,793.1	16	37.4	1.56 (0.70–3.45)	1.63 (0.73–3.61)	2.29 (1.06–4.94)
0.1 to < 10 g/day	4 (3–6)	311,006.7	59	19.0	0.88 (0.45–1.72)	0.92 (0.46–1.81)	0.86 (0.43–1.70)
10 to < 30 g/day	15 (12–21)	338,412.0	89	26.3	1.28 (0.66–2.49)	1.28 (0.66–2.51)	1.31 (0.67–2.57)
30 to < 60 g/day	41 (36–50)	164,704.9	57	34.6	1.62 (0.81–3.20)	1.56 (0.78–3.12)	1.63 (0.81–3.28)
≥ 60 g/day	78 (68–100)	68,908.3	29	42.1	1.70 (0.82–5.96)	1.62 (0.77–3.39)	1.82 (0.87–3.81)
**Women (n = 145,333)**							
Never drinker	0 (0–0)	72,963.2	20	27.4	1.00 (reference)	1.00 (reference)	1.00 (reference)
Former drinker	0 (0–0)	91,852.5	17	18.5	2.56 (1.31–5.05)	2.71 (1.37–5.35)	3.74 (1.96–7.11)
0.1 to < 10 g/day	3 (2–4)	411,646.5	55	13.4	1.66 (0.96–2.87)	1.59 (0.92–2.75)	1.68 (0.97–2.91)
10 to < 20 g/day	13 (10–14)	70,328.0	15	21.3	3.10 (1.53–6.26)	2.93 (1.44–5.95)	2.28 (1.03–5.05)
20 to < 40 g/day	26 (21–31)	31,339.3	5	16.0	2.37 (0.87–6.47)	2.23 (0.81–6.16)	2.64 (0.96–7.28)
≥ 40 g/day	54 (45–72)	15,554.7	2	12.9	2.08 (0.48–9.05)	2.08 (0.47–9.08)	2.46 (0.56–10.8)

*P* = 0.257 for the overall interaction between sex and alcohol consumption categories for cancer mortality (multivariable model).

^a^Estimated from Cox proportional hazard models using age as a timescale to estimate hazard ratios (HRs) and 95 percent confidence intervals (95% CIs). Multivariable model was adjusted for age (timescale), sex (only for total subjects), center, year of screening exam, smoking status, total energy intake, physical activity, BMI, education level, history of diabetes, history of hypertension, history of cardiovascular disease, and family history of cancer.

^b^Estimated from Cox proportional hazard models with alcohol consumption, smoking status, physical activity, total energy intake, and BMI as time-dependent categorical variables and baseline age, sex, center, year of screening exam, education level, history of diabetes, history of hypertension, history of cardiovascular disease, and family history of cancer as time-fixed variables.

*BMI* body mass index, *CI* confidence interval, *HR* hazard ratio, *IQR* interquartile range.

Because cancer mortality is associated with smoking, which correlated with alcohol intake, we performed analyses among never smokers to address the residual confounding by smoking. The association between alcohol consumption and cancer mortality was similar when the analyses were restricted to never smokers (Supplementary Table 6). Also, when we used the competing risk method to analyze mortality while accounting for mortality from causes other than cancer-related disease (Supplementary Table 7), the association between alcohol consumption and cancer mortality was slightly attenuated among men and women, but the results remained qualitatively similar. Both higher frequency and higher quantity were associated with a higher risk of cancer-related mortality in a dose-dependent manner (Supplementary Table 8).

In 3-year lag time analyses by excluding cancer mortality cases that occurred within the first 3 years to deal with possibility in which cancer was present but undiagnosed, we found similar trends (Supplemental Table 9). When we also performed stratified analysis by age (< 40 years old vs ≥ 40 years old), there was no significant interaction on the association between drinking category and cancer mortality, but the association tended to be stronger in older individuals (age ≥ 40 years old vs. age < 40 years old) (Supplemental Table 10).

The participants with no information on alcohol were more likely to be older, women and less educated (Supplementary Table 11). When those with no information on alcohol were included as a separate category, unknown status of alcohol consumption was associated with an increased risk of cancer mortality compared with never drinkers (Supplementary Table 12).

## Discussion

This cohort study of young and middle-aged men and women found a positive association between alcohol consumption and the risk of cancer mortality. When lifetime abstinence history was considered, alcohol consumption was positively associated with cancer mortality in a dose-dependent manner, even with light drinking. This association remained significant after adjusting for potential confounders and even when changes in alcohol consumption and confounders during follow-up were treated as time-varying covariates. Our findings support a linear and independent relationship between the level of alcohol consumption and cancer mortality. Given that low-volume drinking is very common, even a modest increase in cancer risk will have a significant public health impact.

Some cohort studies and meta-analyses have reported a J-shaped relationship between alcohol consumption and cancer risk, with the lowest risk in the light drinking category, whereas other studies have shown that even light drinking is associated with an increased risk of some cancers, especially alcohol-related cancers^3^. A meta-analysis of 18 cohort studies showed a J-shaped relationship between alcohol consumption and all cancer mortality, indicating an inverse association at light drinking (≤ 12.5 g/day), no significant association at moderate drinking (12.6 to 49.9 g/day), and a positive association at heavy alcohol drinking (≥ 50 g/day) compared with non/occasional drinkers^6^. However, most previous cohort studies were subject to methodological issues such as “abstainer bias” (former drinkers misclassified as non-drinkers) and insufficient adjustment for potential confounders. A recent meta-analysis of 87 studies by Stockwell et al. also replicated the J-shaped relationship between alcohol consumption and all-cause mortality, finding a protective effect of low-volume drinking^11^. However, they reported that no significant reduction in mortality risk was observed in studies without abstainer bias that properly adjusted for potential confounders^11^. Similarly, when we did not consider lifetime abstinence history, we also replicated a protective effect in the light drinking category with a J-shape association, but when we analyzed never drinkers and former drinkers separately, we found a positive association between alcohol drinking and cancer mortality, with the increased risk beginning at low-level alcohol consumption. A large prospective cohort study among U.S. adults demonstrated a protective effect of light drinking on cancer mortality even using lifetime abstainers as the referent category, but that association was pronounced among women, middle-aged and older populations, non-Hispanic white subjects, and never or former smokers^31^. The reasons for the differences between that study by Xi et al. and our study are unclear, but the different findings could be attributable to differences in study design, study population (age, ethnicity, socioeconomic status, and comorbidity), alcohol drinking patterns, and the control of potential confounding factors. Our study population was much younger than that in previous studies, with a mean age of 38.9 (SD 9.4) years^32^. Moreover, alcohol drinking patterns and alcohol-related morbidity and mortality can differ by socioeconomic status^14,33–35^. Because the present cohort study included educated, relatively young adults who participated in a regular health checkup program, the subjects of our study had a relatively homogeneous socio-economic status. Alcohol consumption and heavy drinking tends to decline with age^36^ but we consistently observed a linear and positive association between alcohol consumption and cancer mortality when we accounted for variations in alcohol consumption over time, including the baseline and follow-up periods.

Racial and ethnic differences or different drinking patterns might also explain the differences between our findings and those of the previous study^37^. Individuals with *ALDH2* gene variant, a very common genotype among East Asians (including up to 30% of Koreans), can be more susceptible than wild-type to the detrimental effects of alcohol intake^38–40^. Many studies have suggested a role for the *ALDH2* polymorphism and its interaction with alcohol consumption in the development of some cancers^39^. Because we did not have information about the presence of the *ALDH2* genotype, we could not confirm those results, but the effect of drinking on cancer mortality might differ by genotype. Further studies that consider genetic differences in ethanol metabolism are needed to clarify the relationship between light drinking and cancer mortality.

Although the mechanisms responsible for alcohol-associated cancer mortality are not fully understood, potential mechanisms include the following: acetaldehyde, a toxic metabolite of alcohol, has a carcinogenic effect through DNA damage^41^. Ethanol can also stimulate carcinogenesis by inhibiting DNA methylation and interacting with retinoid metabolism^41^. Other possible mechanisms include increased estrogen concentration (mainly for breast cancers), increased levels of plasma insulin-like growth factors produced by the liver following alcohol consumption, alcohol acting as a solvent for tobacco carcinogens, alcohol-related lesions that favor the absorption of carcinogens in the aerodigestive tract epithelium, the production of reactive oxygen and nitrogen species, changes in folate metabolism, and changes in DNA repair^42,43^. Ethanol might be immunosuppressive, thereby enhancing or facilitating carcinogenesis in various organs^43,44^.

## Limitations of this study

We acknowledge the limitations of our study. First, in our study, information on alcohol intake was obtained with self-administered structured questionnaires without information on carbohydrate-deficient transferrin or phosphatidylethanol, objective measures of alcohol intake^45^. Second, different types of alcoholic beverages might play a differential influence on health outcomes but this information was not available^46,47^. Therefore, the possibility of unmeasured or residual confounding factors cannot be excluded. Furthermore, the detailed information on drinking history before baseline status of former and current drinkers except the lifetime abstinence was unavailable, limiting our ability to evaluate the effect of lifetime drinking history on cancer mortality. Third, the relatively short-term follow-up and our use of data about cancer mortality without information about cancer incidence could be another limitation. The cancer mortality that occurred during the relatively short-term follow-up in our study might reflect more aggressive and fatal cancers rather than indolent cancers. Finally, the subjects analyzed in this study were relatively young and highly educated Korean men and women who received health-screening exams, mainly as a routine part of work-related health checkup programs. We compared our study population with a representative sample of the general Korean population (the Korea National Health and Nutrition Examination Survey (KNHANES), and age and sex standardization was performed using the direct method for the age structure of the Korean population aged 20–80 in year 2010. The age and sex standardized prevalence of hypertension (defined as SBP ≥ 140 mmHg or DBP ≥ 90 mmHg or the use of antihypertensive medication), type 2 diabetes (defined as fasting serum glucose level ≥ 126 mg/dL or the use of blood glucose lowering agents), obesity (BMI ≥ 25 kg/m2), and current smoker was lower than those of the general population as previously described^48^, indicating that our study population may be healthier than the general Korean population. Additionally, when we compared cancer mortality rate between the present study population and national statistics, the age and sex standardized mortality from cancer was lower than those of the general population with a standardized mortality ratio of 0.23. Thus, our findings might limit generalizability to other populations with different demographics, or other race/ethnicity.

Despite these potential limitations, our study has major strengths, such as our use of never drinkers as the reference group, the incorporation of changes in alcohol drinking status and confounders as time-varying covariates, control of potential confounding variables^18,19^ and the relative homogeneity of the participants, which might have minimized potential confounding by socio-economic status and accessibility to health care.

## Conclusion

In this large cohort study of young and middle-aged adults free of cancer at baseline, low levels of alcohol consumption were significantly associated with an increased risk of cancer mortality in both men and women. This association was consistently observed after adjustment for potential confounders, even in never-smokers, indicating a linear and independent relationship between alcohol consumption and cancer mortality. Our study findings reaffirm that there is no safe level of alcohol consumption in relation to cancer mortality.

## Supplementary Information

Supplementary Information.

## Abbreviations

ALDH: Aldehyde dehydrogenase
ALT: Alanine aminotransferase
AST: Aspartate aminotransferase
BMI: Body mass index
BP: Blood pressure
CI: Confidence interval
CVD: Cardiovascular disease
GGT: Gamma-glutamyl transpeptidase
HDL-C: High-density lipoprotein-cholesterol
HEPA: Health-enhancing physical activity
HOMA-IR: Homeostasis model assessment of insulin resistance
HR: Hazard ratio
hsCRP: High sensitive C-reactive protein
IPAQ: International physical activity questionnaire
LDL-C: Low-density lipoprotein cholesterol
