# Supplementary material for: Low-level alcohol consumption and cancer mortality
Source: Sci Rep. 2021 Feb 25;11:4585. doi: 10.1038/s41598-021-84181-1 (PMC7907072; doi:10.1038/s41598-021-84181-1)

**Supporting information**

Low-Level Alcohol Consumption and Cancer Mortality

Hyeonyoung Ko^1,2,*^; Yoosoo Chang^3,4,5,*^; Han-Na Kim^6^; Jae-Heon Kang^1^; Hocheol Shin^1,3^; Eunju Sung^1,3,†^; Seungho Ryu^3,4,5,†^

^1^ Department of Family Medicine, Kangbuk Samsung Hospital, Sungkyunkwan University School of Medicine, Seoul, Republic of Korea

^2^ Samsung Seocho Medical Clinic, Kangbuk Samsung Hospital, Sungkyunkwan University School of Medicine, Seoul, Republic of Korea

^3^ Center for Cohort Studies, Total Healthcare Center, Kangbuk Samsung Hospital, Sungkyunkwan University, School of Medicine, Seoul, Republic of Korea

^4^ Department of Occupational and Environmental Medicine, Kangbuk Samsung Hospital, Sungkyunkwan University, School of Medicine, Seoul, Republic of Korea

^5^ Department of Clinical Research Design & Evaluation, SAIHST, Sungkyunkwan University, Seoul, Republic of Korea

^6^ Medical Research Institute, Kangbuk Samsung Hospital, Sungkyunkwan University School of Medicine, Seoul, Republic of Korea

**^*^Hyeonyoung Ko and Yoosoo Chang contributed equally as co-first authors.**

**^†^Seungho Ryu and Eunju Sung contributed equally as co-corresponding authors.**

**^†^Corresponding Authors**:

**Seungho Ryu, MD, PhD**, Department of Occupational and Environmental Medicine, Kangbuk Samsung Hospital, Sungkyunkwan University School of Medicine, Samsung Main Building B2, 250 Taepyung-ro 2ga, Jung-gu 04514, Seoul, Republic of Korea

Tel: +82-2-2001-5137; Fax: +82-2-757-0436; E-mail: [sh703.yoo@gmail.com](mailto:sh703.yoo@gmail.com)

and

**Eunju Sung, MD, PhD**, Department of Family Medicine, Kangbuk Samsung Hospital, Sungkyunkwan University School of Medicine, 29 Saemunan-ro, Jongno-gu, Seoul 03181, Republic of Korea

Tel: +82-2-2001-1790; Fax: +82-2-2001-1404; E-mail: [eju.sung@samsung.com](mailto:choyk2004.cho@samsung.com)

**Running title:** Alcohol consumption and cancer mortality

**Table of Contents**

**Supplementary Methods**

**Supplementary Table 1.** Baseline characteristics according to each center.

**Supplementary Table 2.** Baseline characteristics according to drinking status among men (n = 186,651).

**Supplementary Table 3.** Baseline characteristics according to drinking status among women (n = 145,333).

**Supplementary Table 4.** Number of cancer deaths and site-specific cancer mortality by sex and cause of death.

**Supplementary Table 5.** Hazard ratios (95% CI) for alcohol-related cancer and other cancer mortality by alcohol intake category based on both lifetime drinking status and current drinking status.

**Supplementary Table 6.** Hazard ratios (95% CI) for cancer mortality by lifetime drinking status among never smokers (n = 175,546).

**Supplementary Table 7.** Hazard ratios (95% CI) for cancer mortality by drinking status using competing risk analysis (n = 331,984).

**Supplementary Table 8.** Hazard ratios (95% CI) for cancer mortality by drinking pattern after excluding former drinkers (n = 305,428)

**Supplementary Table 9.** Hazard ratios (95% CI) for cancer mortality by drinking status using 3-year lag time analyses after excluding cases that occurred during the first three years of follow-up (n = 331,820).

**Supplementary Table 10.** Hazard ratios (95% CI) ^a^ for cancer mortality according to drinking status by age.

**Supplementary Table 11.** Baseline characteristics according to missing information on alcohol data

**Supplementary Table 12.** Hazard ratios (95% CI) for cancer mortality by drinking status after including 31,780 participant with missing information on alcohol intake (n = 363,764).

**Supplementary Figure 1.** Multivariable-adjusted hazard ratios for cancer mortality.

**Supplementary methods**

**Comparison of baseline characteristics of participants by study center.**

The Kangbuk Samsung Hospital Total Healthcare Center consists of two centers in Seoul and Suwon, which are all parts of Kangbuk Samsung Hospital. When we compared characteristics of participants according to the study center, participants from Seoul center are more likely to be older, male, and highly educated (**Supplementary Table 1**).

Supplementary Table 1. Baseline characteristics according to each center

| Characteristics | Seoul Center | Suwon Center | P value |
| --- | --- | --- | --- |
| Number | 213,471 | 118,513 |  |
| Age (years) ^a^ | 40.0 (9.7) | 36.9 (8.3) | <0.001 |
| Male (%) | 57.3 | 54.3 | <0.001 |
| Current smoker (%) | 23.8 | 23.7 | 0.562 |
| High education level (%)^c^ | 82.6 | 75.6 | <0.001 |
| HEPA (%)^d^ | 16.9 | 16.0 | <0.001 |
| Hypertension (%) | 12.8 | 10.1 | <0.001 |
| Diabetes (%) | 4.2 | 3.0 | <0.001 |
| History of CVD (%) | 1.3 | 1.0 | <0.001 |
| Family history of cancer (%) | 28.0 | 26.3 | <0.001 |
| Obesity (%)^d^ | 28.4 | 28.0 | 0.018 |
| Body mass index (kg/m^2^) ^a^ | 23.3 (3.3) | 23.3 (3.4) | <0.001 |
| Systolic BP (mmHg) ^a^ | 109.2 (13.5) | 110.2 (12.9) | <0.001 |
| Diastolic BP (mmHg) ^a^ | 70.0 (10.0) | 70.5 (10.0) | <0.001 |
| Glucose (mg/dL) ^a^ | 95.3 (15.3) | 95.6 (14.7) | <0.001 |
| Total cholesterol (mg/dL) ^a^ | 194.4 (34.3) | 192.9 (34.4) | <0.001 |
| LDL-C (mg/dL) ^a^ | 120.0 (32.1) | 119.2 (32.0) | <0.001 |
| HDL-C (mg/dL) ^a^ | 58.5 (15.1) | 58.5 (15.3) | 0.595 |
| Triglycerides (mg/dL) ^b^ | 90 (64-134) | 92 (65-138) | <0.001 |
| AST (U/L) ^b^ | 20 (17-25) | 19 (16-24) | <0.001 |
| ALT (U/L) ^b^ | 18 (13-27) | 18 (12-28) | 0.001 |
| GGT (U/L) ^b^ | 21 (14-36) | 20 (13-35) | <0.001 |
| hsCRP (mg/L) ^b^ | 0.4 (0.2-1.0) | 0.4 (0.2-0.9) | 0.023 |
| HOMA-IR ^b^ | 1.13 (0.74-1.69) | 1.28 (0.85-1.89) | <0.001 |
| Total calorie intake (kcal/day) ^b, f^ | 1,558.8 (1,205-1,953.4) | 1,545.4 (1,185.8-1,942.7) | <0.001 |

Data are expressed as ^a^ mean (standard deviation), ^b^ median (interquartile range), or percentage.

^c^ ≥College graduate; ^d^ defined as physical activity that meets either of two criteria: (i) vigorous-intensity activity on three or more days per week accumulating ≥1,500 metabolic equivalent (MET) min/week; or (ii) seven days of any combination of walking, moderate-intensity, or vigorous-intensity activities achieving at least 3,000 MET-min/week; ^e^ body mass index ≥25kg/m^2^

^f^ Among 227,440 participants with plausible estimated energy intake levels (within three standard deviations of the log-transformed mean energy intake).

Abbreviations: ALT, alanine aminotransferase; AST, aspartate aminotransferase; BP, blood pressure; CVD, cardiovascular disease; GGT, gamma-glutamyl transpeptidase; HDL-C, high-density lipoprotein-cholesterol; HEPA, health-enhancing physically active; hsCRP, high sensitivity C-reactive protein; HOMA-IR, homeostasis model assessment of insulin resistance. LDL-C, low-density lipoprotein cholesterol.

Supplementary Table 2. Baseline characteristics according to drinking status among men (n = 186,651)

| Characteristics | Overall | Lifetime drinking status | | | | | |
| --- | --- | --- | --- | --- | --- | --- | --- |
|  |  | Never drinker | Former drinker | 0 to <10 g/day | 10 to <30 g/day | 30 to <60 g/day | ≥60 g/day |
| Number | 186,651 | 3,018 | 8,106 | 62,309 | 66,697 | 32,615 | 13,906 |
| Age (years) a | 39.7(9.2) | 45.4(13.1) | 40.1(9.5) | 38.7(9.1) | 39.4 (8.9) | 40.7 (8.8) | 41.5 (9.3) |
| Current smoker (%) | 38.8 | 20.8 | 21.5 | 29.4 | 41.1 | 51.4 | 54.2 |
| High education level (%)^c^ | 85.4 | 72.8 | 85.7 | 89.3 | 86.5 | 81.8 | 73.8 |
| HEPA (%)^d^ | 18.1 | 18.6 | 18.6 | 16.6 | 17.8 | 19.7 | 22.6 |
| Hypertension (%) | 16.4 | 18.3 | 12.5 | 12.4 | 16.2 | 21.3 | 25.4 |
| Diabetes (%) | 5.2 | 7.8 | 4.6 | 4.0 | 4.9 | 6.7 | 9.1 |
| History of CVD (%) | 1.5 | 3.2 | 1.5 | 1.3 | 1.4 | 1.7 | 2.0 |
| Family history of cancer (%) | 27.9 | 28.0 | 20.7 | 26.8 | 28.2 | 29.5 | 31.3 |
| Obesity (%)^d^ | 39.9 | 36.7 | 37.3 | 34.9 | 40.5 | 45.4 | 49.0 |
| Body mass index (kg/m^2^) ^a^ | 24.5 (3.0) | 24.2 (3.0) | 24.3 (3.1) | 24.2 (3.0) | 24.6 (2.9) | 24.9 (2.9) | 25.1 (3.1) |
| Systolic BP (mmHg) ^a^ | 115.0 (11.7) | 113.9 (11.9) | 112.5 (11.7) | 113.4 (11.3) | 115.2 (11.6) | 117.0 (11.9) | 118.2 (12.0) |
| Diastolic BP (mmHg) ^a^ | 73.9 (9.4) | 72.8 (9.0) | 72.4 (9.1) | 72.4 (9.0) | 74.1 (9.4) | 75.9 (9.7) | 76.8 (9.7) |
| Glucose (mg/dL) ^a^ | 98.2 (16.6) | 98.7 (19.7) | 96.3 (17.0) | 96.3 (14.4) | 98.1 (16.3) | 100.5 (17.8) | 102.8 (20.9) |
| Total cholesterol (mg/dL) ^a^ | 199.0 (34.7) | 195.8 (35.5) | 197.0 (35.2) | 196.5 (34.3) | 199.6 (34.2) | 202.0 (34.9) | 203.2 (36.2) |
| LDL-C (mg/dL) ^a^ | 127.3 (31.6) | 128.0 (32.5) | 126.1 (31.7) | 127.2 (31.3) | 127.8 (31.3) | 127.1 (32.1) | 126.1 (32.7) |
| HDL-C (mg/dL) ^a^ | 53.0 (12.9) | 50.2 (12.0) | 51.7 (12.4) | 52.1 (12.3) | 53.0 (12.9) | 54.4 (13.4) | 55.4 (14.2) |
| Triglycerides (mg/dL) ^b^ | 113 (80-164) | 104 (75-150) | 105 (75-151) | 104(75-149) | 115 (81-165) | 126 (88-183) | 132 (92-194) |
| AST (U/L) ^b^ | 22 (18-27) | 21 (18-26) | 21 (18-27) | 21 (18-26) | 22 (18-27) | 23 (19-29) | 24 (20-31) |
| ALT (U/L) ^b^ | 24(17-34) | 23(17-33) | 23(17-34) | 23(17-33) | 23 (17-34) | 25 (18-36) | 26 (19-38) |
| GGT (U/L) ^b^ | 30 (21-50) | 23 (17-35) | 25 (18-39) | 25 (18-37) | 31 (21-49) | 42 (27-69) | 51 (31-87) |
| hsCRP (mg/L) ^b^ | 0.5 (0.3-1.0) | 0.5 (0.3-1.1) | 0.5 (0.3-1.0) | 0.5 (0.3-1.0) | 0.5 (0.3-1.0) | 0.6 (0.3-1.1) | 0.6 (0.3-1.1) |
| HOMA-IR ^b^ | 1.28 (0.84-1.92) | 1.30 (0.84-1.96) | 1.26 (0.83-1.91) | 1.25 (0.82-1.87) | 1.28 (0.84-1.92) | 1.32 (0.86-1.99) | 1.35 (0.87-2.07) |
| Total calorie intake (kcal/day) ^b, f^ | 1,664 (1,334.5-2065.1) | 1,643.4 (1,303.1-2060) | 1,649.7 (1,319.1-2061.5) | 1,655.0 (1,329.8-2046.4) | 1,654.2 (1,332.6-2048.8) | 1,684.9 (1,344.5-2,089.2) | 1,731.5 (1,359.6-2,187.7) |

Data are expressed as ^a^ mean (standard deviation), ^b^ median (interquartile range), or percentage.

^c^ ≥College graduate; ^d^ defined as physical activity that meets either of two criteria: (i) vigorous-intensity activity on three or more days per week accumulating ≥1,500 metabolic equivalent (MET) min/week; or (ii) seven days of any combination of walking, moderate-intensity, or vigorous-intensity activities achieving at least 3,000 MET-min/week; ^e^ body mass index ≥25kg/m^2^

^f^ Among 125,828 participants with plausible estimated energy intake levels (within three standard deviations of the log-transformed mean energy intake).

Abbreviations: ALT, alanine aminotransferase; AST, aspartate aminotransferase; BP, blood pressure; CVD, cardiovascular disease; GGT, gamma-glutamyl transpeptidase; HDL-C, high-density lipoprotein-cholesterol; HEPA, health-enhancing physically active; hsCRP, high sensitivity C-reactive protein; HOMA-IR, homeostasis model assessment of insulin resistance. LDL-C, low-density lipoprotein cholesterol.

Supplementary Table 3. Baseline characteristics according to drinking status among women (n = 145,333)

| Characteristics | Overall | Lifetime drinking status | | | | | |
| --- | --- | --- | --- | --- | --- | --- | --- |
|  |  | Never drinker | Former drinker | 0 to <10 g/day | 10 to <20 g/day | 20 to <40 g/day | ≥40 g/day |
| Number | 145,333 | 15,437 | 19,009 | 86,001 | 14,824 | 6,684 | 3,378 |
| Age (years) ^a^ | 37.8 (9.5) | 47.3 (12.2) | 36.5 (8.3) | 37.1 (8.4) | 35.8 (8.6) | 35.5 (8.8) | 34.5 (9.0) |
| Current smoker (%) | 3.0 | 1.3 | 1.5 | 2.0 | 5.8 | 10.0 | 16.7 |
| High education level (%)^c^ | 73.2 | 60.5 | 76.2 | 77.7 | 66.6 | 60.0 | 52.9 |
| HEPA (%)^d^ | 14.5 | 16.0 | 12.3 | 14.2 | 16.1 | 16.6 | 17.5 |
| Hypertension (%) | 5.9 | 16.2 | 4.5 | 4.5 | 4.9 | 5.8 | 6.5 |
| Diabetes (%) | 1.9 | 5.3 | 1.6 | 1.5 | 1.5 | 1.7 | 1.7 |
| History of CVD (%) | 0.8 | 2.3 | 0.7 | 0.6 | 0.6 | 0.7 | 0.9 |
| Family history of cancer (%) | 26.8 | 32.0 | 23.7 | 27.0 | 25.6 | 25.0 | 25.5 |
| Obesity (%)^d^ | 13.2 | 19.1 | 12.7 | 11.7 | 14.5 | 16.6 | 17.7 |
| Body mass index (kg/m^2^) ^a^ | 21.7 (3.1) | 22.4 (3.2) | 21.6 (3.2) | 21.5 (3.0) | 21.9 (3.2) | 22.1 (3.3) | 22.2 (3.4) |
| Systolic BP (mmHg) ^a^ | 102.6 (11.8) | 107.4 (14.2) | 100.7 (11.3) | 102.0 (11.3) | 102.9 (11.4) | 104.0 (11.7) | 104.9 (11.5) |
| Diastolic BP (mmHg) ^a^ | 65.4 (8.6) | 67.5 (9.2) | 64.4 (8.4) | 64.9 (8.3) | 65.9 (8.7) | 66.9 (9.0) | 67.7 (9.0) |
| Glucose (mg/dL) ^a^ | 91.9 (12.0) | 94.9 (15.6) | 90.5 (11.2) | 91.5 (11.3) | 92.0 (12.0) | 92.9 (12.7) | 93.2 (12.4) |
| Total cholesterol (mg/dL) ^a^ | 187.2 (32.8) | 195.5 (35.7) | 186.4 (32.4) | 186.0 (32.3) | 186.3 (32.2) | 187.0 (32.4) | 186.3 (31.9) |
| LDL-C (mg/dL) ^a^ | 109.9 (29.9) | 120.4 (33.1) | 108.9 (29.0) | 109.4 (29.2) | 107.2 (29.5) | 105.9 (30.1) | 102.6 (29.7) |
| HDL-C (mg/dL) a | 65.5 (15.0) | 61.9 (14.7) | 64.2 (14.5) | 65.5 (14.6) | 67.8 (15.5) | 69.5 (16.5) | 71.6 (17.3) |
| Triglycerides (mg/dL) b | 70 (54-96) | 81 (60-113) | 69 (53-95) | 69 (54-93) | 69 (54-95) | 72 (55-100) | 75 (57-104) |
| AST (U/L) ^b^ | 17 (15-21) | 19 (16-23) | 17 (15-20) | 17 (15-20) | 17 (15-20) | 28 (15-21) | 18 (15-22) |
| ALT (U/L) ^b^ | 13 (10-17) | 15 (11-20) | 13 (10-17) | 13 (10-17) | 13 (10-17) | 13 (10-17) | 13 (10-18) |
| GGT (U/L) ^b^ | 13 (10-18) | 14 (11-19) | 12 (10-17) | 13 (10-17) | 14 (11-19) | 16 (12-22) | 17 (13-26) |
| hsCRP (mg/L) ^b^ | 0.3 (0.2-0.7) | 0.4 (0.2-0.9) | 0.3 (0.2-0.7) | 0.3 (0.2-0.6) | 0.3 (0.2-0.7) | 0.3 (0.2-0.7) | 0.3 (0.2-0.7) |
| HOMA-IR ^b^ | 1.07 (0.72-1.57) | 1.13 (0.73-1.69) | 1.08 (0.72-1.58) | 1.06 (0.71-1.54) | 1.07 (0.71-1.57) | 1.09 (0.73-1.62) | 1.12 (0.74-1.66) |
| Total calorie intake (kcal/day) ^b, f^ | 1,403.9 (1,049.1-1,787.0) | 1,438.3 (1090.6-1,797.5) | 1,378.7 (1,016.7-1,781.9) | 1,417.9 (1,069.9-1,796.0) | 1,354.1 (1,002.9-1,737.6) | 1,335.9 (958.1-1750.1) | 1,345.3 (936.0-1,810.8) |

Data are expressed as ^a^ mean (standard deviation), ^b^ median (interquartile range), or percentage.

^c^ ≥College graduate; ^d^ defined as physical activity that meets either of two criteria: (i) vigorous-intensity activity on three or more days per week accumulating ≥1,500 metabolic equivalent (MET) min/week; or (ii) seven days of any combination of walking, moderate-intensity, or vigorous-intensity activities achieving at least 3,000 MET-min/week; ^e^ body mass index ≥25kg/m^2^

^f^ Among 101,612 participants with plausible estimated energy intake levels (within three standard deviations of the log-transformed mean energy intake).

Abbreviations: ALT, alanine aminotransferase; AST, aspartate aminotransferase; BP, blood pressure; CVD, cardiovascular disease; GGT, gamma-glutamyl transpeptidase; HDL-C, high-density lipoprotein-cholesterol; HEPA, health-enhancing physically active; hsCRP, high sensitivity C-reactive protein; HOMA-IR, homeostasis model assessment of insulin resistance. LDL-C, low-density lipoprotein cholesterol.

Supplementary Table 4. Number of cancer deaths and site-specific cancer mortality by sex and cause of death.

|  | Men  (n = 186,651 ; 940,222.3 PY) | | Women  (n = 145,333 ; 693,684.1 PY) | |
| --- | --- | --- | --- | --- |
|  | Number of deaths | Mortality rate per 100,000 person-years | Number of deaths | Mortality rate per 100,000 person-years |
| Malignant neoplasms (C00-C97, D00-D48) | 260 | 27.7 (24.5-31.2) | 114 | 16.4 (13.7-19.7) |
| Digestive cancer (C15-C26) | 114 | 12.1 (10.1-14.6) | 53 | 6.9 (5.8-10.0) |
| Stomach cancer (C16) | 18 | 1.9 (1.2-3.0) | 16 | 2.3 (1.4-3.8) |
| Colorectal cancer (C18-C20) | 10 | 1.1 (0.6-2.0) | 6 | 0.9 (0.4-1.9) |
| Hepatobiliary cancer (C22.0-C24.9) | 50 | 5.3 (4.0-7.0) | 13 | 1.9 (1.1-3.2) |
| Pancreatic cancer (C25) | 28 | 3.0 (2.1-4.3) | 17 | 2.5 (1.5-3.9) |
| Lung cancer (C33, C34) | 75 | 8.0 (6.4-10.0) | 14 | 2.0 (1.2-3.4) |
| Breast cancer (C50) | 1 | 0.1 (0.01-0.8) | 10 | 1.4 (0.8-2.7) |
| Genitourinary (C64-C68) | 7 | 0.7 (0.4-1.6) | 2 | 0.3 (0.1-1.2) |
| Blood cancer (C81-C96) | 27 | 2.9 (2.0-4.2) | 11 | 1.6 (0.9-2.9) |

The type of cancer was classified according to the International Classification of Diseases and Related Health Problems 10^th^ Revision.

Abbreviations: PY, person-years.

Supplementary Table 5**.** Hazard ratios (95% CI) for alcohol-related cancer and other cancer mortality by alcohol intake category based on both lifetime drinking status and current drinking status.

| Alcohol intake category | Multivariable-adjusted HR (95% CI) ^a^ | |
| --- | --- | --- |
|  | Alcohol-related cancer mortality | Other cancer mortality |
| Total (N=331,984) |  |  |
| Lifetime abstainer | 1.00 (reference) | 1.00 (reference) |
| Current abstainer | 5.55 (1.97-15.63) | 2.90 (1.68-5.02) |
| 0.1 to <10 g/day | 2.10 (0.82-5.36) | 1.37 (0.86-2.18) |
| 10 to <20 g/day | 3.08 (1.09-8.70) | 1.85 (1.09-3.17) |
| 20 to <40 g/day | 2.93 (1.01-8.52) | 2.19 (1.27-3.77) |
| ≥40 g/day | 3.51 (1.20-10.25) | 2.32 (1.32-4.06) |
| Men (n= 186,651) |  |  |
| Lifetime abstainer | 1.00 (reference) | 1.00 (reference) |
| Current abstainer | 2.66 (0.504.13) | 2.21 (0.89-5.48) |
| 0.1 to <10 g/day | 0.98 (0.21-4.51) | 0.90 (0.40-2.04) |
| 10 to <30 g/day | 1.52 (0.34-6.77) | 1.29 (0.58-2.88) |
| 30 to <60 g/day | 2.12 (0.47-9.69) | 1.55 (0.68-3.55) |
| ≥60 g/day | 2.18 (0.44-10.79) | 1.54 (0.63-3.75 |
| Women (n=145,333) |  |  |
| Lifetime abstainer | 1.00 (reference) | 1.00 (reference) |
| Current abstainer | 5.25 (1.37-20.09) | 2.24 (1.08-4.65) |
| 0.1 to <10 g/day | 2.00 (0.61-6.57) | 1.23 (0.68-2.23) |
| 10 to <20 g/day | 1.87 (0.32-10.80) | 1.71 (0.72-4.08) |
| 20 to <40 g/day | 2.26 (0.23-21.75) | 3.00 (1.15-7.84) |
| ≥40 g/day | - | 2.32 (0.52-10.30) |

^a^ Estimated from Cox proportional hazard models using age as a timescale to estimate hazard ratios (HRs) and 95 percent confidence intervals (95% CIs). Multivariable model was adjusted for age (timescale), sex (only for total subjects), center, year of screening exam, smoking status, total energy intake, physical activity, BMI, education level, history of diabetes, history of hypertension, history of cardiovascular disease, and family history of cancer.

Supplementary Table 6. Hazard ratios (95% CI) for cancer mortality by lifetime drinking status among never smokers (n = 175,546).

| Alcohol consumption category | | Multivariable-adjusted HR (95% CI) ^a^ | | |  |
| --- | --- | --- | --- | --- | --- |
|  |  | Total (N = 175,546) | Men (n = 58,110) | Women (n = 117,436) | |
| Total/women | Men |  |  |  | |
| Never drinker | Never drinker | 1.00 (reference) | 1.00 (reference) | 1.00 (reference) | |
| Former drinker | Former drinker | 2.42 (1.18-4.95) | 0.92 (0.18-4.80) | 3.01 (1.36-6.64) | |
| 0 to <10 g/day | 0 to <10 g/day | 1.85 (1.04-3.27) | 0.98 (0.28-3.52) | 1.87 (0.98-3.56) | |
| 10 to <20 g/day | 10 to <30 g/day | 2.88 (1.44-5.80) | 1.39 (0.39-4.97) | 3.53 (1.54-8.07) | |
| 20 to <40 g/day | 30 to <60 g/day | 2.52 (1.08-5.84) | 1.44 (0.33-6.31) | 2.38 (0.66-8.53) | |
| ≥40 g/day | ≥60 g/day | 3.13 (1.26-7.79) | 1.38 (0.22-8.58) | 3.67 (0.81-16.60) | |

^a^ Estimated from Cox proportional hazard models using age as a timescale to estimate hazard ratios (HRs) and 95 percent confidence intervals (95% CIs). Multivariable model was adjusted for age (timescale), sex (only for total subjects), center, year of screening exam, smoking status, total energy intake, physical activity, body mass index, education level, history of diabetes, history of hypertension, history of cardiovascular disease, and family history of cancer.

Abbreviations: CI, confidence interval; HR, hazard ratio.

Supplemental Table 7. Hazard ratios (95% CI) for cancer mortality by drinking status using competing risk analysis (n = 331,984).

| Alcohol consumption category | | Sub-distribution hazard model (95% CI) ^a^ | | |  |
| --- | --- | --- | --- | --- | --- |
|  |  | Total (N = 331,984) | Men (n = 186,651) | Women (n = 145,333) | |
| Total/women | Men |  |  |  | |
| Never drinker | Never drinker | 1.00 (reference) | 1.00 (reference) | 1.00 (reference) | |
| Former drinker | Former drinker | 2.88 (1.72-4.82) | 1.62 (0.74-3.56) | 2.71 (1.37-5.39) | |
| 0 to <10 g/day | 0 to <10 g/day | 1.69 (1.10-2.60) | 0.92 (0.47-1.80) | 1.59 (0.94-2.70) | |
| 10 to <20 g/day | 10 to <30 g/day | 2.39 (1.44-3.94) | 1.29 (0.66-2.50) | 2.94 (1.49-5.80) | |
| 20 to <40 g/day | 30 to <60 g/day | 2.55 (1.54-4.24) | 1.56 (0.78-3.09) | 2.24 (0.83-6.06) | |
| ≥40 g/day | ≥60 g/day | 2.72 (1.62-4.57) | 1.61 (0.77-3.35) | 2.06 (0.47-9.00) | |

^a^ Fine and Gray proportional hazard models with age as a timescale were used to estimate hazard ratios (HRs) and 95 percent confidence intervals (95% CIs). Multivariable model was adjusted for age (timescale), sex (only for total subjects), center, year of screening exam, smoking status, total energy intake, physical activity, body mass index, education level, history of diabetes, history of hypertension, history of cardiovascular disease, and family history of cancer.

Abbreviations: CI, confidence interval.

Supplementary Table 8. Hazard ratios (95% CI) for cancer mortality by drinking pattern after excluding former drinkers (n = 305,428)

| Drinking pattern | Multivariable-adjusted HR (95% CI) ^*^ | | |
| --- | --- | --- | --- |
|  | Total (n = 305,428) | Men (n = 126,707) | Women (n = 178,721) |
| Frequency of drinking (drinks/week) |  |  |  |
| 0 | 1.00 (reference) | 1.00 (reference) | 1.00 (reference) |
| 1-2 | 1.68 (1.13-2.51) | 1.02 (0.55-1.87) | 1.64 (0.97-2.78) |
| 3-4 | 1.60 (0.99-2.58) | 1.02 (0.53-1.95) | 1.22 (0.41-3.63) |
| 5-6 | 2.85 (1.67-4.86) | 1.73 (0.87-3.43) | 1.45 (0.19-10.93) |
| 7 | 3.49 (1.84-6.64) | 2.11 (0.98-4.56) | - |
| *P* for trend | <.001 | .005 | .384 |
| Number of drinks consumed per drinking day |  |  |  |
| 0 | 1.00 (reference) | 1.00 (reference) | 1.00 (reference) |
| 1-2 | 1.64 (1.07-2.51) | 0.95 (0.48-1.87) | 1.41 (0.80-2.47) |
| 3-5 | 1.75 (1.12-2.73) | 1.01 (0.54-1.89) | 1.49 (0.79-2.83) |
| ≥6 | 2.55 (1.61-4.04) | 1.48 (0.79-2.76) | 3.33 (1.63-6.81) |
| *P* for trend | <.001 | .015 | .004 |

^*^ Estimated from Cox proportional hazard models using age as a timescale to estimate hazard ratios (HRs) and 95 percent confidence intervals (95% CIs). Multivariable model was adjusted for age (timescale), sex (only for total subjects), center, year of screening exam, smoking status, total energy intake, physical activity, body mass index, education level, history of diabetes, history of hypertension, history of cardiovascular disease, and family history of cancer.

CI = confidence interval; HR = hazard ratio.

Supplementary Table 9. Hazard ratios (95% CI) for cancer mortality by drinking status using 3-year lag time analyses after excluding cases that occurred during the first three years of follow-up (n = 331,820).

| Alcohol consumption category | | Multivariable-adjusted HR (95% CI) ^a^ | | |
| --- | --- | --- | --- | --- |
|  |  | Total (N = 331,820) | Men (n = 186,540) | Women (n = 145,280) |
| Total/women | Men |  |  |  |
| Never drinker | Never drinker | 1.00 (reference) | 1.00 (reference) | 1.00 (reference) |
| Former drinker | Former drinker | 1.83 (0.81-4.17) | 1.14 (0.34-3.78) | 1.33 (0.40-4.40) |
| 0.1 to <10 g/day | 0.1 to <10 g/day | 2.01 (1.12-3.62) | 0.95 (0.37-2.48) | 1.92 (0.90-4.09) |
| 10 to <20 g/day | 10 to <30 g/day | 2.94 (1.52-5.67) | 1.35 (0.53-3.47) | 3.71 (1.45-9.47) |
| 20 to <40 g/day | 30 to <60 g/day | 3.14 (1.61-6.14) | 1.80 (0.68-4.72) | 1.62 (0.34-7.76) |
| ≥40 g/day | ≥60 g/day | 3.18 (1.60-6.34) | 1.94 (0.70-5.33) | 1.93 (0.24-15.70) |

^a^ Estimated from Cox proportional hazard models using age as a timescale to estimate hazard ratios (HRs) and 95 percent confidence intervals (95% CIs). Multivariable model was adjusted for age (timescale), sex (only for total subjects), center, year of screening exam, smoking status, total energy intake, physical activity, body mass index, education level, history of diabetes, history of hypertension, history of cardiovascular disease, and family history of cancer.

Abbreviations: CI, confidence interval; HR, hazard ratio.

Supplementary Table 10. Hazard ratios (95% CI) ^a^ for cancer mortality according to drinking status by age.

| Age | Drinking status | | | | | | *P* for interaction |
| --- | --- | --- | --- | --- | --- | --- | --- |
|  | Never drinker | Former drinker | 0 to <10 g/day | 10 to <20 g/day | 20 to <40 g/day | ≥40 g/day |  |
| <40 years (n = 199,160) | reference | 1.50 (0.17-13.45) | 1.72 (0.23-12.64) | 1.05 (0.13-8.58) | 1.46 (0.18-11.99) | 1.59 (0.19-13.40) | .205 |
| ≥40 years (n = 132,824) | reference | 3.10 (1.82-5.26) | 1.57 (1.01-2.44) | 2.61 (1.60-4.25) | 2.68 (1.62-4.42) | 2.84 (1.70-4.74) |  |

^a^ Estimated from Cox proportional hazard models using age as a timescale to estimate hazard ratios (HRs) and 95 percent confidence intervals (95% CIs). Multivariable model was adjusted for age (timescale), sex, center, year of screening exam, smoking status, total energy intake, physical activity, body mass index, education level, history of diabetes, history of hypertension, history of cardiovascular disease, and family history of cancer.

Abbreviations: HEPA, health-enhancing physically active.

Supplementary Table 11. Baseline characteristics according to missing information on alcohol data

| Characteristics | Alcohol data available | Alcohol data missing | P value |
| --- | --- | --- | --- |
| Number | 331,984 | 31,780 |  |
| Age (years) ^a^ | 38.9 (9.4) | 48.0 (11.5) | <0.001 |
| Male (%) | 56.2 | 23.0 | <0.001 |
| Current smoker (%) | 23.8 | 8.9 | <0.001 |
| High education level (%)^c^ | 80.0 | 55.8 | <0.001 |
| HEPA (%)^d^ | 16.6 | 18.8 | <0.001 |
| Hypertension (%) | 11.8 | 17.8 | <0.001 |
| Diabetes (%) | 3.8 | 6.6 | <0.001 |
| History of CVD (%) | 1.2 | 2.8 | <0.001 |
| Family history of cancer (%) | 27.4 | 32.5 | <0.001 |
| Obesity (%)^d^ | 28.2 | 25.1 | <0.001 |
| Body mass index (kg/m^2^) ^a^ | 23.3 (3.3) | 23.0 (3.2) | <0.001 |
| Systolic BP (mmHg) ^a^ | 109.6 (13.3) | 109.5 (14.2) | 0.244 |
| Diastolic BP (mmHg) ^a^ | 70.2 (10.0) | 69.5 (9.7) | <0.001 |
| Glucose (mg/dL) ^a^ | 95.4 (15.1) | 96.9 (17.6) | <0.001 |
| Total cholesterol (mg/dL) ^a^ | 193.9 (34.4) | 196.2 (36.0) | <0.001 |
| LDL-C (mg/dL) ^a^ | 119.7 (32.1) | 121.9 (33.1) | <0.001 |
| HDL-C (mg/dL) ^a^ | 58.5 (15.2) | 59.3 (14.9) | <0.001 |
| Triglycerides (mg/dL) ^b^ | 91 (64-136) | 87 (63-125) | <0.001 |
| AST (U/L) ^b^ | 20 (16-25) | 20 (16-24) | <0.001 |
| ALT (U/L) ^b^ | 18 (13-27) | 17 (13-24) | <0.001 |
| GGT (U/L) ^b^ | 21 (13-36) | 16 (12-26) | <0.001 |
| hsCRP (mg/L) ^b^ | 0.4 (0.2-0.9) | 0.4 (0.2-1.0) | 0.422 |
| HOMA-IR ^b^ | 1.18 (0.78-1.77) | 1.12 (0.74-1.71) | <0.001 |
| Total calorie intake (kcal/day) ^b, f^ | 1,553.7 (1,197.9-1,949.4) | 1,536.9 (1,204.9-1,907.7) | 0.530 |

Data are expressed as ^a^ mean (standard deviation), ^b^ median (interquartile range), or percentage.

^c^ ≥College graduate; ^d^ defined as physical activity that meets either of two criteria: (i) vigorous-intensity activity on three or more days per week accumulating ≥1,500 metabolic equivalent (MET) min/week; or (ii) seven days of any combination of walking, moderate-intensity, or vigorous-intensity activities achieving at least 3,000 MET-min/week; ^e^ body mass index ≥25kg/m^2^

^f^ Among 241,356 participants with plausible estimated energy intake levels (within three standard deviations of the log-transformed mean energy intake).

Abbreviations: ALT, alanine aminotransferase; AST, aspartate aminotransferase; BP, blood pressure; CVD, cardiovascular disease; GGT, gamma-glutamyl transpeptidase; HDL-C, high-density lipoprotein-cholesterol; HEPA, health-enhancing physically active; hsCRP, high sensitivity C-reactive protein; HOMA-IR, homeostasis model assessment of insulin resistance. LDL-C, low-density lipoprotein cholesterol.

Supplementary Table 12. Hazard ratios (95% CI) for cancer mortality by drinking status after including 31,780 participant with missing information on alcohol intake (n = 363,764).

| Alcohol consumption category | | Multivariable-adjusted HR  (95% CI) ^a^ | | |
| --- | --- | --- | --- | --- |
|  |  | Total (N = 363,764) | Men (n = 193,963) | Women (n = 169,801) |
| Total/women | Men |  |  |  |
| Never drinker | Never drinker | 1.00 (reference) | 1.00 (reference) | 1.00 (reference) |
| Former drinker | Former drinker | 2.42 (1.46-4.03) | 1.59 (0.72-3.53) | 2.47 (1.26-4.84) |
| 0 to <10 g/day | 0 to <10 g/day | 1.42 (0.93-2.17) | 0.89 (0.45-1.75) | 1.47 (0.85-2.52) |
| 10 to <20 g/day | 10 to <30 g/day | 1.80 (1.13-2.88) | 1.22 (0.63-2.38) | 2.69 (1.34-5.40) |
| 20 to <40 g/day | 30 to <60 g/day | 1.89 (1.18-3.05) | 1.45 (0.73-2.88) | 2.08 (0.76-5.67) |
| ≥40 g/day | ≥60 g/day | 1.98 (1.22-3.22) | 1.50 (0.72-3.13) | 1.93 (0.44-8.42) |
| Unknown | Unknown | 1.78 (1.17-2.72) | 1.55 (0.77-3.09) | 1.62 (0.94-2.79) |

^a^ Estimated from Cox proportional hazard models using age as a timescale to estimate hazard ratios (HRs) and 95 percent confidence intervals (95% CIs). Multivariable model was adjusted for age (timescale), sex (only for total subjects), center, year of screening exam, smoking status, total energy intake, physical activity, BMI, education level, history of diabetes, history of hypertension, history of cardiovascular disease, and family history of cancer.

Abbreviations: CI, confidence interval.

Supplemental figure 1. Multivariable-adjusted hazard ratios for cancer mortality. Curves represent adjusted hazard ratios for cancer mortality based on restricted cubic splines with knots at the 5^th^, 27.5^th^, 50^th^, 72.5^th^, and 95^th^ percentiles of alcohol consumption distribution. Models were adjusted for age, sex, center, year of screening exam, smoking status, total energy intake, physical activity, body mass index, education level, history of diabetes, history of hypertension, history of cardiovascular disease, and family history of cancer.


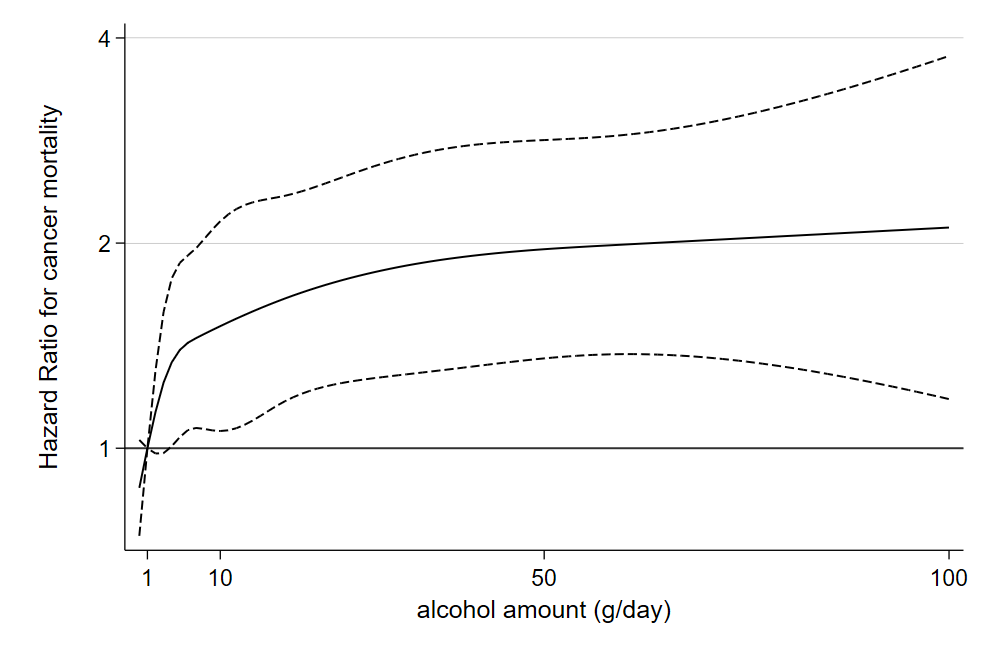

Supplement: Supplementary file 1 — Supplementary Information. [file 41598_2021_84181_MOESM1_ESM.docx]
